# Image Privacy Protection Communication Scheme by Fibonacci Interleaved Diffusion and Non-Degenerate Discrete Chaos

**DOI:** 10.3390/e27080790

**Published:** 2025-07-25

**Authors:** Zhiyu Xie, Weihong Xie, Xiyuan Cheng, Zhengqin Yuan, Wenbin Cheng, Yiting Lin

**Affiliations:** 1 School of Electronic Information, University of Electronic Science and Technology of China Zhongshan Institute, Zhongshan 528402, China; zhiyuxie@ieee.org (Z.X.); 2022010002053@stu.zsc.edu.cn (W.X.); zhengqinyuancs@gmail.com (Z.Y.);; 2 School of Information and Communication Engineering, University of Electronic Science and Technology of China, Chengdu 611731, China; 3 School of Automation, Guangdong University of Technology, Guangzhou 510006, China; 2112304365@mail2.gdut.edu.cn

**Keywords:** nonlinear dynamics, image encryption, privacy-preserving, information security, cryptography

## Abstract

The rapid development of network communication technology has led to an increased focus on the security of image storage and transmission in multimedia information. This paper proposes an enhanced image security communication scheme based on Fibonacci interleaved diffusion and non-degenerate chaotic system to address the inadequacy of current image encryption technology. The scheme utilizes a hash function to extract the hash characteristic values of the plaintext image, generating initial perturbation keys to drive the chaotic system to generate initial pseudo-random sequences. Subsequently, the input image is subjected to a light scrambling process at the bit level. The *Q* matrix generated by the Fibonacci sequence is then employed to diffuse the obtained intermediate cipher image. The final ciphertext image is then generated by random direction confusion. Throughout the encryption process, plaintext correlation mechanisms are employed. Consequently, due to the feedback loop of the plaintext, this algorithm is capable of resisting known-plaintext attacks and chosen-plaintext attacks. Theoretical analysis and empirical results demonstrate that the algorithm fulfils the cryptographic requirements of confusion, diffusion, and avalanche effects, while also exhibiting a robust password space and excellent numerical statistical properties. Consequently, the security enhancement mechanism based on Fibonacci interleaved diffusion and non-degenerate chaotic system proposed in this paper effectively enhances the algorithm’s resistance to cryptographic attacks.

## 1. Introduction

In recent years, the exponential growth of computer communication technology and network technology has led to a significant increase in the frequency, extent, and speed of data and information transmission via networks [1,2,3,4]. This has led to the emergence of new requirements for a secure transmission environment. Among the various types of data exchanged for information exchange, images present a particularly sensitive case, as they contain a significant amount of valuable information [5,6,7]. Consequently, the utilisation of image encryption technology [8,9,10] can effectively prevent the leakage of crucial data during the transmission process. A plethora of encryption methodologies have been proposed, including image steganography [11,12,13], biometric encoding [14,15], semi-tensor product [16,17,18], bit-level encryption [9,19,20], chaos theory [9,21,22,23] and others [24,25,26,27]. Among these, the unpredictability, pseudo-randomness, and high sensitivity to chaotic initial values make it the most effective and widely used method for image encryption algorithms [28,29,30,31,32].

From an international perspective, numerous scholars have achieved a series of significant theoretical and applied results in the utilization of chaotic systems for image encryption [33,34,35,36]. In existing research on chaotic image encryption, the performance of chaotic systems and algorithms has a significant impact on the security and efficiency of cryptographic systems [19,37,38]. On the other hand, image encryption methods based on Fibonacci have also gradually gained attention. In 2021, Ref. [39] proposed a novel image encryption scheme based on Quasigroup and Fibonacci transform. This scheme employs a novel image encryption structure that can encrypt images of any size. Experimental results have demonstrated the superiority of this scheme. In 2023, Ref. [40] presented a secure image encryption method using Fibonacci and Tribonacci transforms. Experimental results demonstrate that the proposed image encryption algorithm can securely resist various illegal attacks. In 2024, Ref. [41] introduced a reversible fragile watermark model. This model first generates a watermark from the image using DCT transform and then encrypts the watermark using Fibonacci *Q* matrix technology to enhance the security of the model. Despite some progress in digital image encryption research in this area, the algorithms designed still have certain limitations due to inherent flaws in encryption systems [21,42]. From a security perspective, existing pixel-level chaotic image encryption algorithms still require further improvement to combat various illegal attacks. Continued research in this field is of great significance for protecting user data privacy [43,44,45], ensuring data integrity, and defending against various forms of attacks [46,47,48]. It is expected to provide more innovative solutions for the development of information security [49,50,51] and secure digital image transmission.

This paper presents a novel image privacy protection scheme based on Fibonacci interleaved diffusion and non-degenerate discrete chaos. Experimental results show that the algorithm exhibits excellent encryption effectiveness and good efficiency. The proposed image encryption algorithm is able to effectively resist various illegal attacks. The main contributions and innovations of the paper are as follows:

- This encryption algorithm introduces a novel diffusion mechanism. By leveraging the principles of Fibonacci-related mathematics, a Fibonacci sequence interleaved diffusion method is devised, which effectively resists statistical analysis and enhances the security of encryption.
- The majority of existing encryption algorithms are susceptible to potential risks. This image privacy protection scheme employs plaintext correlation to generate dynamic chaotic keys, significantly enhancing the ability to resist cryptographic attacks.
- A significant number of the encryption algorithms currently in use are considered to be unreasonable. In the absence of relevant plaintext or ciphertext feedback, they become highly vulnerable to known-plaintext or chosen-plaintext attacks. In order to address this issue, the proposed secure image encryption scheme employs a dynamic feedback mechanism to continuously update encryption keys based on encrypted data. Building upon our current foundation of cryptanalysis research [52,53,54], it enhances security and strengthens the ability to withstand attacks like chosen-plaintext and chosen-ciphertext attacks.

The following section outlines the structure of the remainder of this paper. Section 2 provides an introduction to robust discrete hyper-chaotic systems and the rules of Fibonacci. Section 3 introduces the encryption algorithm designed in this paper. Section 4 presents the experimental and simulation results. The final section presents the conclusion of the paper.

## 2. Related Theory

### 2.1. Non-Degenerate Chaotic System

The non-degenerate discrete-time chaotic system [55] was selected for this study due to its simplicity, ease of implementation, and demonstrated chaotic properties. The chaotic system was derived step-by-step from the following formula.

In order to obtain the matrix Ma, which is the result of a transformation of the matrix *T*, an asymptotically stable nominal system matrix *T* is required initially. This is achieved by applying a similar transformation to the matrix *T* using the non-singular matrix *N*.

(1) $$Ma=NTN^{-1}=\left(\begin{array}{ccc} 0.6500 & 0.1500 & -0.1500\\ 0.3300 & 0.4700 & -0.3300\\ 0.1800 & -0.1800 & 0.3200 \end{array}\right)$$

Consequently, the iterative equation of the system is expressed as follows:

(2) $$\left\{\begin{array}{c} X\left(k+1\right)={Ma}_{11}X\left(k\right)+{Ma}_{12}Y\left(k\right)+{Ma}_{13}Z\left(k\right)\\ Y\left(k+1\right)={Ma}_{21}X\left(k\right)+{Ma}_{22}Y\left(k\right)+{Ma}_{23}Z\left(k\right)\\ Z\left(k+1\right)={Ma}_{31}X\left(k\right)+{Ma}_{32}Y\left(k\right)+{Ma}_{33}Z\left(k\right) \end{array}\right.$$

The poles of the nominal system are defined by the unit matrix and the uniform bounded inverse controller. The results of the designed discrete-time chaotic system are presented in Equation (3).

(3) $$\begin{array}{cc} \left(\begin{array}{c} X(k+1)\\ Y(k+1)\\ Z(k+1) \end{array}\right) & ={Ma}_{3\times 3}\left(\begin{array}{c} X(k)\\ Y(k)\\ Z(k) \end{array}\right)+\left(\begin{array}{ccc} 1 & 0 & 0\\ 0 & 1 & 0\\ 0 & 0 & 1 \end{array}\right)\left(\begin{array}{c} \;\mathrm{mod}\;\left(\sigma X(k),\varepsilon \right)\\ \;\mathrm{mod}\;\left(\sigma Y(k),\varepsilon \right)\\ \;\mathrm{mod}\;\left(\sigma Z(k),\varepsilon \right) \end{array}\right) \end{array}$$

The final mathematical formulation of the chaotic system employed in this study is presented below:

(4) $$\left\{\begin{array}{c} X\left(k+1\right)=Ma_{11}X\left(k\right)+Ma_{12}Y\left(k\right)+Ma_{13}Z\left(k\right)+\mathrm{mod}\left(\sigma X(k),\varepsilon \right)\\ Y\left(k+1\right)=Ma_{21}X\left(k\right)+Ma_{22}Y\left(k\right)+Ma_{23}Z\left(k\right)+\mathrm{mod}\left(\sigma Y(k),\varepsilon \right)\\ Z\left(k+1\right)=Ma_{31}X\left(k\right)+Ma_{32}Y\left(k\right)+Ma_{33}Z\left(k\right)+\mathrm{mod}\left(\sigma Z(k),\varepsilon \right) \end{array}\right.$$

### 2.2. Fibonacci Q Matrix

The Fibonacci Sequence is a sequence that the mathematician Fibonacci studied using the study of rabbit reproduction as an example, hence its designation as the “Rabbit Sequence”, also known as the Golden Section Sequence. It attracted considerable interest from society as soon as it was proposed. Following research, it was discovered that this sequence, which is characterized by a seemingly magical quality, plays an immeasurably important role. In particular, the sequence begins with 0 and 1, with each subsequent item being the sum of the previous two items. This can be expressed mathematically as follows:

(5) $$\left\{\begin{array}{c} F(0)=0\\ F(1)=1\\ F(n)=F(n-1)+F(n-2)\;(n\geq 2) \end{array}\right.$$

This sequence has a wide range of applications in the fields of mathematics and computing, demonstrating some special mathematical properties and patterns. In this paper, the Fibonacci sequence is combined with image encryption to explore a diffusion method based on this mathematical approach. The specific steps are as follows:

The elements of the Fibonacci sequence are denoted as $F_{n}$.

(6) $$F_{n}=F_{n-1}+F_{n-2}\;n⩾3$$

The initial term of the Fibonacci matrix is as follows:

(7) $$Q=\left[\begin{array}{cc} 1 & 1\\ 1 & 0 \end{array}\right]$$

The *n*-th power of the Fibonacci matrix is as follows:

(8) $$Q^{n}=\left[\begin{array}{cc} F_{n+1} & F_{n}\\ F_{n} & F_{n-1} \end{array}\right]$$

The inverse matrix of the *n*-th power of the Fibonacci matrix is as follows:

(9) $$Q^{-n}=\left[\begin{array}{cc} F_{n-1} & -F_{n}\\ -F_{n} & F_{n+1} \end{array}\right]$$

## 3. The Proposed Encryption Algorithm

**Figure 1:**
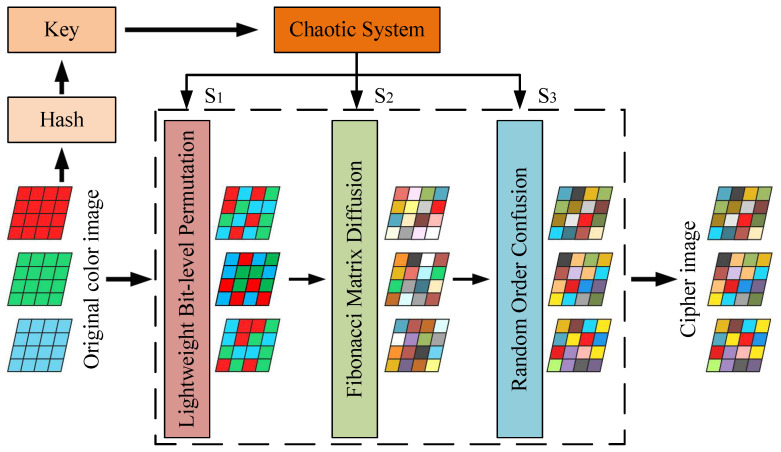
The proposed algorithm flowchart.

In contrast to traditional algorithms, this paper utilizes a discrete hyper-chaotic system and a hash function to generate the chaotic key required for encryption. This enhances the security of the algorithm and increases the difficulty of code breaking. The flowchart depicting the algorithm design is presented in Figure 1.

**Figure 2:**
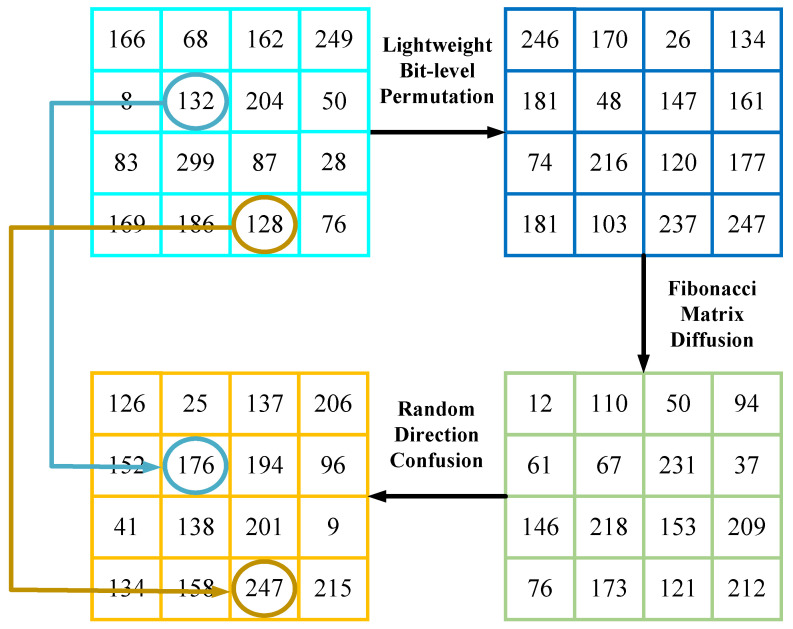
Single-channel encryption example.

Take the matrix as an example to encrypt a single channel, and the specific encryption process is shown in Figure 2.

### 3.1. Chaos Key Generation and Sequence Preprocessing

After extracting features from the plaintext image *P* using a hash function, we obtain character-based fixed-length hexadecimal numbers. To simplify computation, each number is converted to a decimal number and represented by the variable Hash. The image hash is then mathematically processed to obtain the initial key parameters, which are substituted into the chaotic system to obtain the chaotic sequences $S_{1}$, $S_{2}$ and $S_{3}$.

### 3.2. Lightweight Bit-Level Permutation

Image scrambling is a commonly employed encryption technique that aligns with the principles of confusion and diffusion as outlined in Shannon’s cryptography theory. This approach can enhance the security performance of encryption algorithms. There are two principal methods for scrambling images: pixel-level scrambling and bit-level scrambling. In pixel-level scrambling, only the pixel positions of the image are altered, while the statistical histogram of the scrambled image remains unchanged. This makes it susceptible to statistical analysis attacks. Bit-level scrambling not only changes the pixel positions of the image but also alters the pixel grayscale values, thereby providing stronger security performance. However, compared to pixel-level scrambling, efficiency is relatively lower for bit-level scrambling. Some researchers have highlighted that only scrambling image encryption schemes are insecure as they are vulnerable to chosen-plaintext or known-plaintext attacks, requiring a considerable amount of plaintext to decipher at least half of the equivalent passwords. The complexity of attacking encryption algorithms that only involve scrambling operations is proportional to the square of the required plaintext quantity. Based on this, a lightweight bit-level image scrambling method is proposed in this article, and Algorithm 1 introduces the specific operational process.
**Algorithm 1** Lightweight Bit-level Permutation**Require:** Chaotic sequence $S_{1}$; plaintext image *P* of size [height,width,3]
**Ensure:** Intermediate ciphertext image $C_{1}$
 1: Separate RGB channels: PR=P(:,:,1); PG=P(:,:,2); PB=P(:,:,3) 2: $X\leftarrow S_{1}\left(1:height\times 3\right)$;    $Y\leftarrow S_{1}\left(height\times 3+1:height\times 3+width\times 8\right)$ 3: [,indexX]←sort(X);    [,indexY]←sort(Y) 4: Convert channels to binary and reshape:$$\begin{array}{cc} IR2 & \leftarrow \mathrm{reshape}(\mathrm{dec}2\mathrm{bin}(PR),height,width\times 8)\\ IG2 & \leftarrow \mathrm{reshape}(\mathrm{dec}2\mathrm{bin}(PG),height,width\times 8)\\ IB2 & \leftarrow \mathrm{reshape}(\mathrm{dec}2\mathrm{bin}(PB),height,width\times 8) \end{array}$$ 5: Stack binary channels: inputP←[IR2;IG2;IB2] 6: outputP←inputP 7: **for** i=1 to height×3 **do** 8:     **for** j=1 to width×8 **do** 9:         Swap: outputP(i,j)↔outputP(indexX(i),indexY(j))10:     **end for**11: **end for**12: Split permuted matrix:$$\begin{array}{cc} OR & \leftarrow outputP(1:height,:)\\ OG & \leftarrow outputP(height+1:2height,:)\\ OB & \leftarrow outputP(2height+1:3height,:) \end{array}$$13: Reconstruct channels:$$\begin{array}{cc} C_{1}\left(:,:,1\right) & \leftarrow \mathrm{uint}8\left(\mathrm{reshape}\left(\mathrm{bin}2\mathrm{dec}\left(OR\right),height,width\right)\right)\\ C_{1}\left(:,:,2\right) & \leftarrow \mathrm{uint}8\left(\mathrm{reshape}\left(\mathrm{bin}2\mathrm{dec}\left(OG\right),height,width\right)\right)\\ C_{1}\left(:,:,3\right) & \leftarrow \mathrm{uint}8(\mathrm{reshape}(\mathrm{bin}2\mathrm{dec}(OB),height,width)) \end{array}$$


Step 1: Expand the RGB components of the color plaintext image *P* with dimensions M×N×3 bit by bit, each with a length of M×8N, represented as Pr, Pg and Pb. Combine the RGB components in top-to-bottom order to form a binary matrix of dimensions M×8N×3, denoted as $P_{rgb}$.

Step 2: Process the chaotic sequence $S_{1}$ to generate index sequences rowK and colK. These are used for row and column scrambling of the bit image. By swapping the rows and columns of the image bit matrix, the scrambled bit matrix $P^{\prime }rgb$ is obtained.

Step 3: Resize the scrambled bit matrix for subsequent diffusion operations. The size should be changed from the original 3M×8N back to M×N×3, resulting in the scrambled image denoted as $C_{1}$.

### 3.3. Fibonacci Matrix Diffusion

The RGB layering of the permuted image $C_{1}$ is performed, after which a selection of different Fibonacci number sequence matrices from the chaotic sequence $S_{2}$ is made, with the intention of diffusing each layer of $C_{1}$. This results in the diffused image $C_{2}$. The Fibonacci diffusion algorithm is shown in Algorithm 2, with specific operational steps as follows:
**Algorithm 2** Fibonacci Matrix Diffusion**Require:** Chaotic matrix FN; intermediate image $C_{1}$ of size M×N
**Ensure:** Diffused ciphertext image $C_{2}$
 1: $R\leftarrow \mathrm{double}(C_{1})$ 2: **for** i=1 to *M* step 2 **do** 3:     **for** j=1 to *N* step 2 **do** 4:         q←FN(i,j) 5:         $Q\leftarrow \left[\begin{array}{cc} F(q+1) & F(q)\\ F(q) & F(q-1) \end{array}\right]$ 6:         $C_{x}\leftarrow R\left(i:i+1,j:j+1\right)$ 7:         $C_{2}\left(i:i+1,j:j+1\right)\leftarrow C_{x}\times Q$ 8:     **end for** 9: **end for**10: $C_{2}\leftarrow \mathrm{mod}\left(C_{2},\;256\right)$


Step 1: Reconstruct $S_{2}$ into a chaotic matrix of the same size as $C_{1}$, and then preprocess it into integers within the range of [3:21] as follows:

(10) $$\left\{\begin{array}{c} x=reshape(\left(100\times S_{2}\left(1:M\times N\right)\right),\left[M,N\right])\\ FN=round(mod(x,17)+3) \end{array}\right.$$

where *M* and *N* represent the width and height of the image respectively.

Step 2: Substitute the obtained chaotic matrix FN into the Fibonacci *Q* matrix constructed by Formula (8) to obtain the matrix $S^{n}$.

(11) $$S^{n}=\left[\begin{array}{cc} F(FN(i,j)+1) & F(FN(i,j))\\ F(FN(i,j)) & F(FN(i,j)-1) \end{array}\right]$$

where i=(1,3,…M−1); j=(1,3,…N−1).

Step 3: Substitute the intermediate ciphertext image $C_{1}$ into the Fibonacci *Q* matrix constructed by Formula (8) to obtain the matrix $C_{x}$:

(12) $$C_{x}=\left[\begin{array}{cc} C_{1}\left(i,j\right) & C_{1}\left(i,j+1\right)\\ C_{1}\left(i+1,j\right) & C_{1}\left(i+1,j+1\right) \end{array}\right]$$

Step 4: Diffuse the two matrices $S^{n}$ and $C_{x}$ obtained block by block.

(13) $$fz=S^{n}\times Cx$$

Step 5: The diffused matrix is the ciphertext matrix *C*.

(14) $$\left\{\begin{array}{c} C(i,j)=fz(1,1)\\ C(i,j+1)=fz(1,2)\\ C(i+1,j)=fz(2,1)\\ C(i+1,j+1)=fz(2,2) \end{array}\right.$$

Step 6: Place the values of the ciphertext matrix *C* in the appropriate range, where $C_{2}$ is the diffused ciphertext image.

(15) $$C_{2}=\mathrm{mod}\left(C,256\right)$$

### 3.4. Random Direction Confusion

Convert the chaotic sequence $S_{3}$ to a chaotic index matrix *I* of the same size as the intermediate ciphertext $C_{2}$, then perform $2^{32}$ power operations on the index matrix *I* to obtain the operation matrix *S*. The specific operations are shown in Algorithm 3, and the calculation formula is as follows:

(16) $$\;\left\{\begin{array}{cc} C_{3}\left(I\left(x,y\right),y\right)=mod\left(C_{2}\left(I\left(x,y\right),y\right)+C_{2}\left(I\left(M,N\right),N\right)+S\left(I\left(x,y\right),y\right),256\right) & \mathrm{for}\;i=1,j=1\\ C_{3}\left(I\left(x,y\right),y\right)=mod\left(C_{2}\left(I\left(x,y\right),y\right)+C_{3}\left(I\left(x-1,N\right),N\right)+S\left(I\left(x,y\right),y\right),256\right) & \mathrm{for}\;i=2∼W,j=1\\ C_{3}\left(I\left(x,y\right),y\right)=mod\left(C_{2}\left(I\left(x,y\right),y\right)+C_{3}\left(I\left(x,y-1\right),y-1\right)+S\left(I\left(x,y\right),y\right),256\right) & \mathrm{for}\;i=1∼W,j=2∼W \end{array}\right.$$

**Algorithm 3** Random Direction Confusion**Require:** Computing matrix *S*; index matrix *I*; intermediate ciphertext $C_{2}$ of size M×N
**Ensure:** Final ciphertext $C_{3}$
 1: **for** x=1 to *M* **do** 2:     **for** y=1 to *N* **do** 3:         **if** y=1 **then** 4:            **if** x=1 **then** 5:                $C_{3}\left(I\left(x,y\right),y\right)\leftarrow \mathrm{mod}\left(C_{2}\left(I\left(x,y\right),y\right)+C_{2}\left(I\left(M,N\right),N\right)+S\left(I\left(x,y\right),y\right),\;256\right)$ 6:            **else** 7:                $C_{3}\left(I\left(x,y\right),y\right)\leftarrow \mathrm{mod}\left(C_{2}\left(I\left(x,y\right),y\right)+C_{3}\left(I\left(x-1,N\right),N\right)+S\left(I\left(x,y\right),y\right),\;256\right)$ 8:            **end if** 9:         **else**10:            $C_{3}\left(I\left(x,y\right),y\right)\leftarrow \mathrm{mod}\left(C_{2}\left(I\left(x,y\right),y\right)+C_{3}\left(I\left(x,y-1\right),y-1\right)+S\left(I\left(x,y\right),y\right),\;256\right)$11:         **end if**12:     **end for**13: **end for**


**Figure 3:**
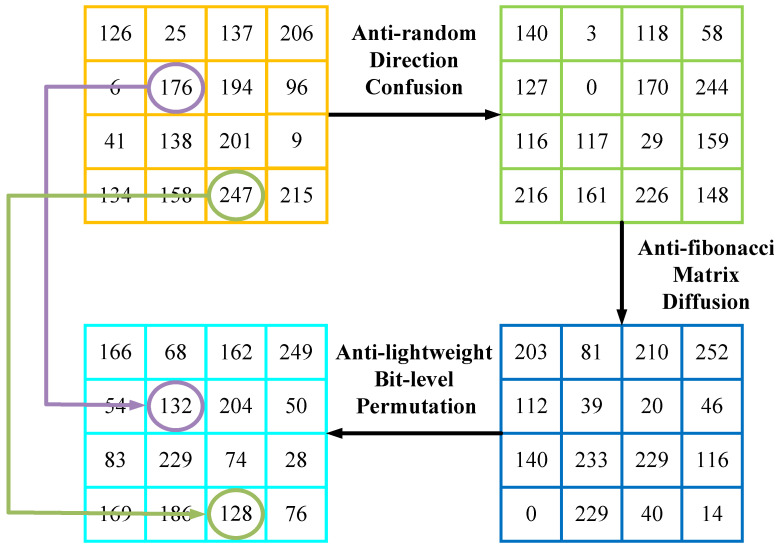
Single-channel decryption example.

The encryption process is hereby completed, and $C_{3}$ is the final ciphertext image. The single-channel decryption flow is shown in Figure 3.

## 4. Experimental Results and Analysis Discussion

The experimental platform used was a MacBook Pro with MATLAB R2022b laboratory software installed. The device is equipped with a 2.2 GHz quad-core Intel Core i7 processor, 16 GB of RAM, and runs on the Windows 10 operating system. The images presented in this paper are sourced from the USC-SIPI database.

### 4.1. Histogram Analysis

**Figure 4:**
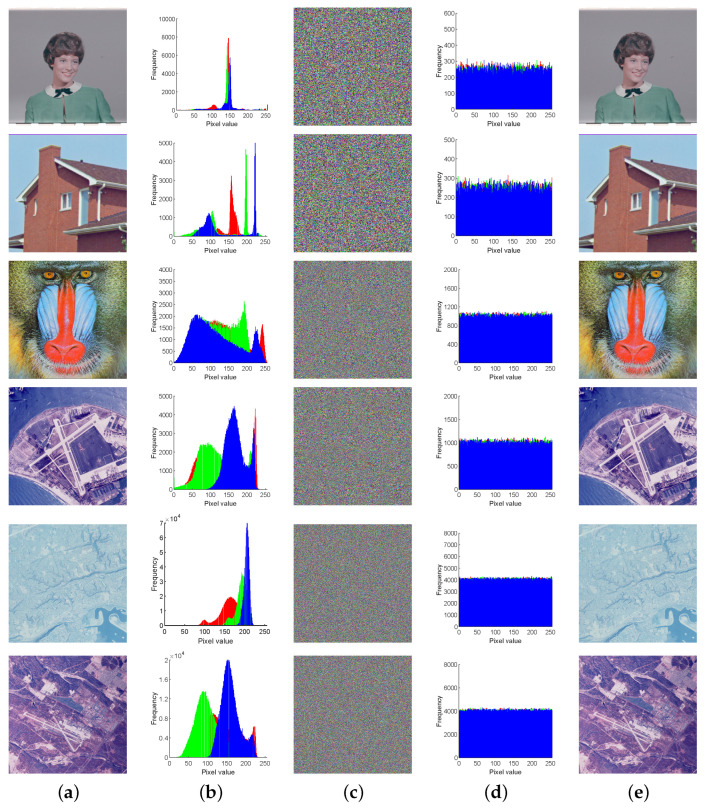
Plaintext and ciphertext images and corresponding histograms. (**a**) Plain-images. (**b**) Histograms of (**a**). (**c**) Encryption results of (**a**). (**d**) Histograms of (**c**). (**e**) Decryption results of (**c**).

The histogram illustrates the distribution of each gray level in the image and its corresponding frequency. Generally, the histogram of a plaintext image displays specific statistical patterns, while the histogram of an encrypted image exhibits statistical characteristics similar to a noise distribution. Therefore, effective encryption algorithms can transform the image into a form resembling a noise distribution to conceal the primary information of the image. One plaintext image of each size was selected for histogram testing, as shown in Figure 4. The encrypted image effectively conceals the main information of the plaintext image, preventing attackers from cracking the ciphertext image through statistical analysis.

### 4.2. The Coefficient of Adjacent Pixels

**Figure 5:**
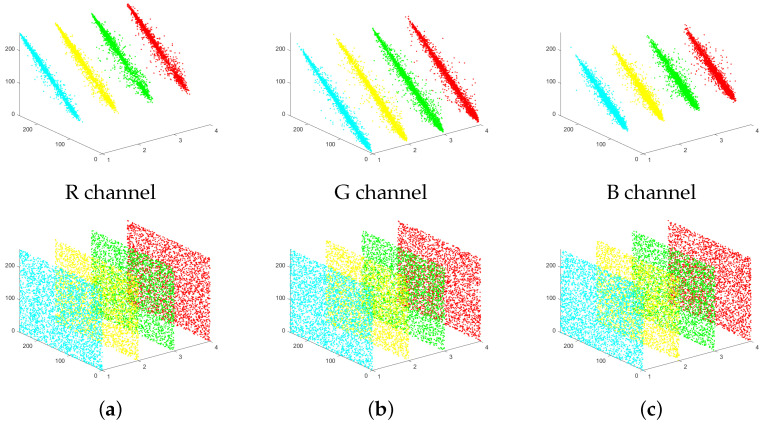
Adjacent pixels’ correlation of plaintext image and ciphertext image. (**a**) R channel; (**b**) G channel; (**c**) B channel.

The objective of image encryption algorithms is to break the correlation between pixels. This prevents attackers from relying on pixel correlation for decryption attempts. This paper uses the ‘Lena’ image as an example to illustrate this process. We randomly selected 3000 pairs of neighboring pixels from the plaintext and ciphertext. Then, we calculated the correlation coefficients of these neighboring pixels in horizontal, vertical, diagonal, and anti-diagonal directions. The scatter plots corresponding to the experiments are displayed in Figure 5, and the results of the correlation analysis are presented in Table 1. The specific calculation formula is as follows:

(17) $$\left\{\begin{array}{c} r_{xy}=\frac{cov(x,y)}{\sqrt{D(x)}\sqrt{D(y)}}\\ \mathrm{cov}\left(x,y\right)=\frac{1}{N}\sum_{i=1}^{N}\left(x_{i}-E\left(x\right)\right)\left(y_{i}-E\left(y\right)\right)\\ D\left(x\right)=\frac{1}{N}\sum_{i=1}^{N}{\left(x_{i}-E\left(x\right)\right)}^{2}\\ E\left(x\right)=\frac{1}{N}\sum_{i=1}^{N}x_{i} \end{array}\right.$$

where $x_{i}$ and $y_{i}$ constitute the *i*-th pair of horizontal, vertical, diagonal or anti-diagonal neighboring pixels, N is the total number of horizontal/vertical/diagonal/anti-diagonal neighboring pixels, cov(x,y) is the covariance between pixel values *x* and *y*, D(x) and D(y) are the pixel value *x* and pixel value *y* mean-square error, and E(x) and E(y) are the expected values of pixel value *x* and pixel value *y*, respectively. $r_{xy}$ is the correlation coefficient of pixel values *x* and *y*.

The findings demonstrate that our encryption algorithm successfully minimizes the correlation between pixels, rendering it almost undetectable in the ciphertext. This provides strong evidence that our proposed encryption algorithm is highly secure.

### 4.3. Differential Attack Analysis

To evaluate the robustness of the proposed image encryption algorithm against differential attacks, we analyze three key metrics: the Number of Pixels Change Rate (NPCR), the Unified Average Changing Intensity (UACI), and the Bit-level Average Change Intensity (BACI). These metrics are commonly used to measure the sensitivity of the ciphertext to small changes in the plaintext and help determine whether the encryption scheme can effectively resist differential cryptanalysis.

**NPCR** measures the percentage of pixels that change value when a single pixel in the plaintext is altered. A higher NPCR value indicates stronger diffusion characteristics in the encryption algorithm. NPCR is computed as follows:

(18) $$NPCR=\frac{1}{M\times N}\sum_{i=1}^{M}\sum_{j=1}^{N}D\left(i,j\right)\times 100\%$$

where M×N is the image size, and D(i,j) is defined as follows:

(19) $$D\left(i,j\right)=\left\{\begin{array}{cc} 0, & C_{1}\left(i,j\right)=C_{2}\left(i,j\right)\\ 1, & C_{1}\left(i,j\right)\neq C_{2}\left(i,j\right) \end{array}\right.$$

Here, $C_{1}$ and $C_{2}$ denote the encrypted images generated from two plaintexts differing by a single pixel.

**UACI** evaluates the average intensity of differences between the ciphertexts, defined by the following:

(20) $$UACI=\frac{1}{M\times N}\sum_{i=1}^{M}\sum_{j=1}^{N}\frac{|C_{1}\left(i,j\right)-C_{2}\left(i,j\right)|}{255}\times 100\%$$

A higher UACI value indicates better resistance to differential attacks by ensuring greater changes in pixel intensity.

**BACI** extends UACI to the bit level, capturing average bit-wise changes between two ciphertexts:

(21) $$BACI=\frac{1}{8\times M\times N}\sum_{i=1}^{M}\sum_{j=1}^{N}\mathrm{Hamming}\left(C_{1}\left(i,j\right),C_{2}\left(i,j\right)\right)\times 100\%$$

where Hamming(·,·) calculates the number of differing bits between corresponding pixel values.

**Figure 6:**
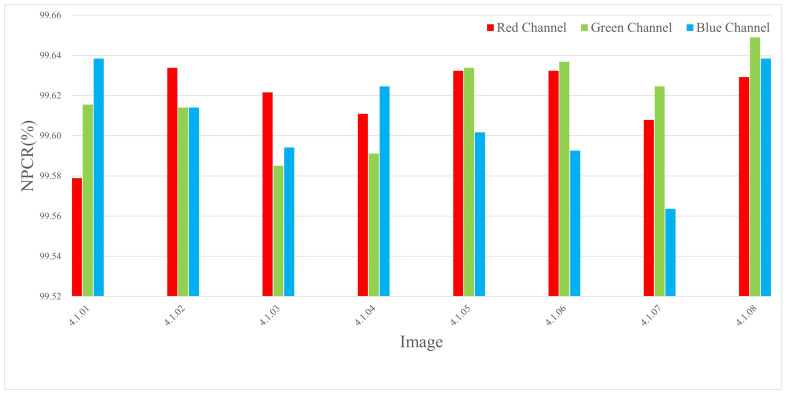
The NPCR test values for different channels of 256 × 256 size image.

**Figure 7:**
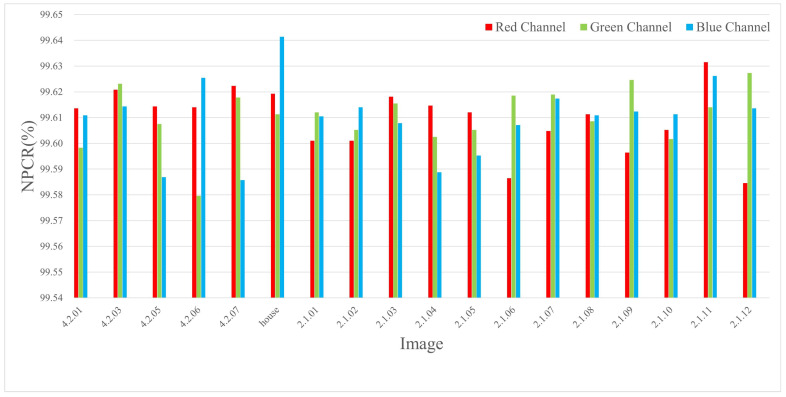
The NPCR test values for different channels of 512 × 512 size image.

**Figure 8:**
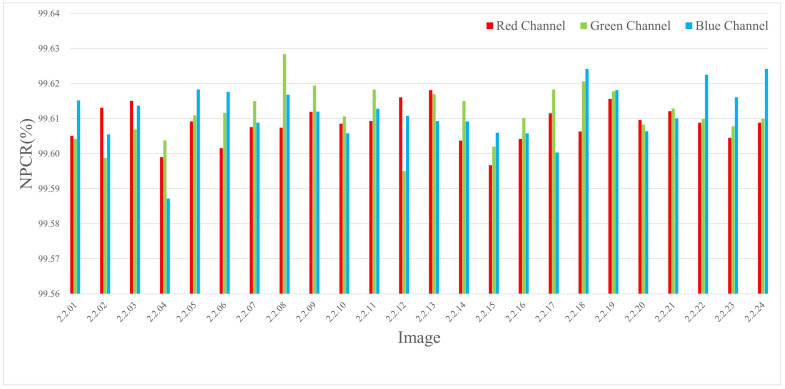
The NPCR test values for different channels of 1024 × 1024 size image.

The experiments were conducted by encrypting images of three different sizes, where a single pixel in the plaintext was modified and the corresponding encrypted outputs were compared. The results are listed in Table 4 and Table 2, and the NPCR visualization comparison results are listed in Figure 6, Figure 7, and Figure 8, respectively. The consistently high NPCR, UACI, and BACI values across different resolutions validate the proposed algorithm’s strong resistance against differential attacks.

### 4.4. Image Quality Analysis

Peak Signal to Noise Ratio (PSNR) and Structural Similarity (SSIM) are frequently used to evaluate the quality of encryption in image processing. PSNR includes Mean Square Error (MSE) as a component, which is defined as follows:

(22) $$\left\{\begin{array}{c} \mathrm{MSE}=\frac{1}{H\times W}\sum_{i=1}^{H}\sum_{j=1}^{W}{\left(X\left(i,j\right)-Y\left(i,j\right)\right)}^{2}\\ \mathrm{PSNR}=10\times \log_{10}\left(\frac{Q^{2}}{MSE}\right) \end{array}\right.$$

where MSE represents the mean square error between the plaintext image *X* and the ciphertext image *Y*, with *H* and *W* representing the height and width of the image, respectively. *Q* denotes the pixel level of the image.

SSIM is a measure of the similarity between two images, defined as follows:

(23) $$\mathrm{SSIM}\left(X,Y\right)=\frac{\left(2\mu_{X}\mu_{Y}+{\left(0.01L\right)}^{2}\right)\left(2\sigma_{XY}+{\left(0.03L\right)}^{2}\right)}{\left(\mu_{X}^{2}+\mu_{Y}^{2}+{\left(0.01L\right)}^{2}\right)\left(\sigma_{X}^{2}+\sigma_{Y}^{2}+{\left(0.03L\right)}^{2}\right)}$$

where $\mu_{X}$ and $\mu_{Y}$ denote the mean values of image *X* and *Y*, respectively. The standard deviation of image *X* and *Y* are denoted by $\sigma_{X}$ and $\sigma_{Y}$, respectively. *L* represents the dynamic range of pixel values.

Equations (22) and (23) are used to calculate the values of MSE, PSNR, and SSIM. Additionally, to ensure generality, several images were selected to test the encryption module. The encrypted images should have a PSNR of less than 10 dB and an SSIM value close to 0. Table 4 and Table 3 details the results of the tests. The experimental results demonstrate the excellent encryption performance of our algorithm.

### 4.5. Information Entropy Analysis

In image encryption, information entropy serves as a vital measure, representing the extent of uncertainty linked to image data and commonly serving to assess the randomness within the system. A higher information entropy value suggests enhanced uncertainty and diminished visibility of image data, indicating better encryption efficacy of the algorithm. To illustrate, a contrast was drawn between the information entropy of the initial and encrypted images. For the information source *m*, the information entropy H(m) can be calculated using the following equation:

(24) $$H\left(m\right)=-\sum_{i=1}^{L}p\left(m_{i}\right)\log_{2}p(m_{i})$$

where *L* is the total number of symbols m(i)∈m and $p(m_{i})$ denotes the probability of the symbols. From Table 4, we can see that the experimental results are close to 8, so the proposed algorithm has good information entropy properties.

### 4.6. Key Space Analysis

Cryptography experts emphasize that to effectively resist brute-force attacks, the key length of chaotic encryption systems should be no less than 128 bits. In the chaotic system used in this paper [55], the key space is defined as

(25) $$S\in \{a_{ij},\delta_{i},\epsilon_{i},x_{i}\left(0\right),\mathrm{Hash}256\},\;i,j=1,2,3,$$

which comprises a total of 55 key parameters, including the nominal system parameters of the non-degenerate hyper-chaotic system, parameters of the feedback controller, initial conditions of four distinct key types, and the SHA-2 hash value.

Based on experimental analysis, the effective key lengths are as follows: the nominal system parameters provide a key space of approximately $10^{16\times 9}\approx 2^{144}$, the feedback controller parameters contribute around $10^{11\times 6}=10^{66}\approx 2^{219}$, the initial chaotic values account for roughly $10^{17+16+15}=10^{48}\approx 2^{159}$, and the SHA-2 hash value itself corresponds to a key length of $2^{256}$. Summing these components yields a total key length of

144+219+159+256=778bits.

### 4.7. Analysis of Plaintext Sensitivity

**Figure 9:**
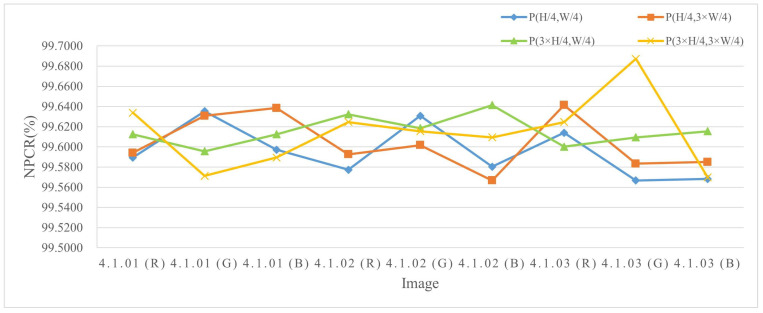
Plaintext sensitivity test results for images of size 256 × 256.

**Figure 10:**
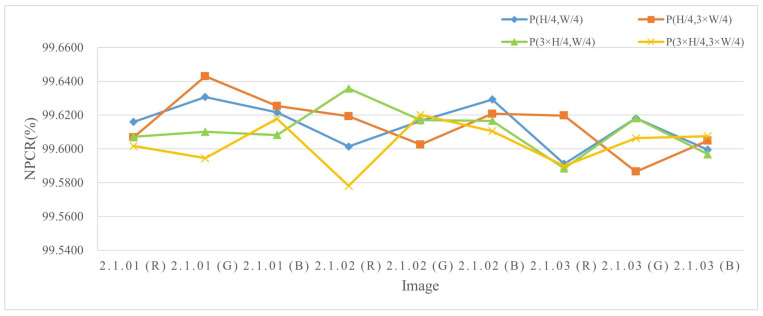
Plaintext sensitivity test results for images of size 512 × 512.

**Figure 11:**
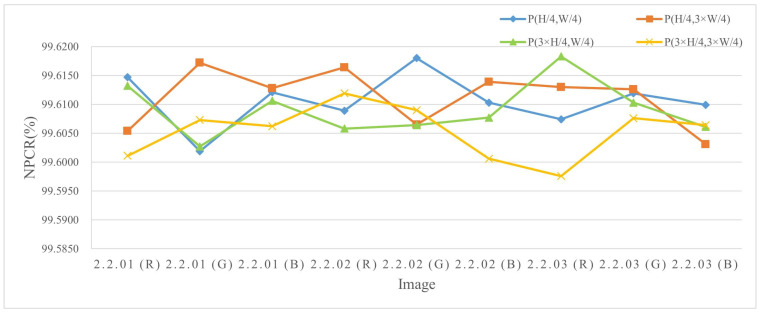
Plaintext sensitivity test results for images of size 1024 × 1024.

The sensitivity of plaintext refers to the extent of ciphertext changes observed when operating on the pixels of plaintext. Insufficient sensitivity of plaintext in encryption algorithms can increase the risk of leakage, and plaintext sensitivity plays an important role in enhancing the algorithm’s ability to resist plaintext attacks. In this section, four pixels of plaintext images are randomly selected and the encryption effects of the original image and the processed plaintext image are compared, with the results shown in Table 5. The experimental results using images of different sizes are shown in Figure 9, Figure 10 and Figure 11. From the experimental data, it can be seen that the algorithm proposed in this research has good plaintext sensitivity.

### 4.8. Theoretical Security Analysis of Fibonacci Diffusion

#### 4.8.1. Effective Nonlinearity Through Dynamic Matrix Diffusion

The proposed diffusion mechanism utilizes a block-wise transformation:

$$C_{x}\mapsto S^{n}\cdot C_{x}\;\mathrm{mod}\;256$$

where $C_{x}$ is a 2×2 pixel block, and $S^{n}$ is a Fibonacci Q-matrix of power *n* (as defined in Equation (8)). Although the transformation is linear per block, the use of a dynamically changing matrix $S^{n}$—with indices determined by the chaotic matrix FN(i,j)—induces system-level nonlinearity.

Two key cryptographic properties are introduced:

- **Exponential Amplification**: Elements of $S^{n}$ follow the Fibonacci recurrence F(n)=F(n−1)+F(n−2), with growth rate $\Theta (\phi^{n})$, where $\phi =\frac{1+\sqrt{5}}{2}\approx 1.618$. As *n* increases, even minor differences in $C_{x}$ are exponentially amplified.
- **Path Confusion**: The dynamic index FN(i,j), derived from a chaotic sequence $S_{2}$, assigns a unique matrix $S^{n}$ to each block, disrupting any attempt at consistent linear modeling across the image.

The scheme therefore achieves confusion in Shannon’s sense, while retaining invertibility via $Q^{-n}$ (Equation (9)).

#### 4.8.2. Resistance to Differential Cryptanalysis

Let $\Delta C_{x}$ be a one-pixel input difference, with ${∥\Delta C_{x}∥}_{1}=1$. The output difference is as follows:

$$\delta =S^{n}\cdot \Delta C_{x}\;\mathrm{mod}\;256$$

We approximate the expected Hamming weight of δ as follows:

$${E[∥\delta ∥}_{1}]\geq \frac{1}{4}\sum_{k=1}^{4}\sum_{j=1}^{4}\left|S_{k,j}^{n}\right|=\Theta \left(\phi^{n}\right)$$

This implies that at least 0.7n bits are expected to change on average. Given n∈[3,21], the minimum expected bit change satisfies the following:

$$\min_{n\geq 3}{E[∥\delta ∥}_{1}]>2.1\;\mathrm{bits}\;\Rightarrow \;\mathrm{NPCR}>99.6\%$$

This theoretical bound corroborates the empirical values reported in Section 4.3, confirming robustness against differential attacks.

#### 4.8.3. Key Sensitivity Analysis

Assuming a one-step tampering of the chaotic index, i.e., $n^{\prime }=n\pm 1$, the deviation in the transformation matrix is quantified by the Frobenius norm:

$$∥S^{n}-S^{n^{\prime }}{∥}_{F}\geq \left|F\left(n+1\right)-F\left(n\right)\right|=F\left(n-1\right)$$

For n≥7, F(n−1)>256. This yields a pixel change probability approximated by an exponential decay model:

$$P\left(\mathrm{pixel}\;\mathrm{change}\right)\geq 1-e^{-F(n-1)/64}>98.2\%$$

This estimation reflects the high diffusion sensitivity to minor key variations. Since FN(i,j) is derived from $S_{2}$ via the following:

$$FN\left(i,j\right)=\mathrm{round}(\mathrm{mod}\left(100\cdot S_{2}\left(i,j\right),17\right)+3)$$

recovering *n* requires inversion of the chaotic map, which for a 128-bit key space implies a brute-force complexity of $\Omega (2^{128})$, ensuring resistance against chosen-plaintext attacks.

#### 4.8.4. Resistance to Algebraic Attacks

The Fibonacci recurrence F(n)=F(n−1)+F(n−2) introduces algebraic structure, which may suggest susceptibility to equation-solving attacks. However, the encryption structure effectively prevents this by the following:

- Each 2×2 block generates four linear equations but includes six unknowns: four matrix entries of $S^{n}$ and two plaintext variables.
- The chaotic index *n* differs per block, preventing consistent coefficient reuse and eliminating possibilities of system-wide equation alignment.
- The modular reduction operation (mod256) introduces nonlinear discontinuities (wrap-around effects), further complicating algebraic inference.

Hence, any system of equations derived from known-plaintext attacks becomes underdetermined, nonuniform, and nonlinear, rendering algebraic cryptanalysis infeasible in practice.

### 4.9. Cryptanalysis of the Proposed Encryption Algorithm

To evaluate the robustness of the proposed Fibonacci-chaos encryption scheme against chosen-plaintext attacks (CPA), we conducted a targeted cryptanalysis experiment based on our previous cryptanalytic research framework [52,53,54]. In a CPA scenario, an adversary can arbitrarily select plaintext images and obtain their corresponding ciphertexts, which is particularly effective in revealing structural weaknesses in diffusion mechanisms. Our methodology employs uniform-color test images—known to expose deterministic patterns in Fibonacci-based diffusion systems.

Four monochromatic images of size 512×512×3 were generated with all pixel values set to the following:

- **170 (10101010)**: Probes alternating bit patterns.
- **255 (11111111)**: Evaluates all-one input handling.

**Figure 12:**
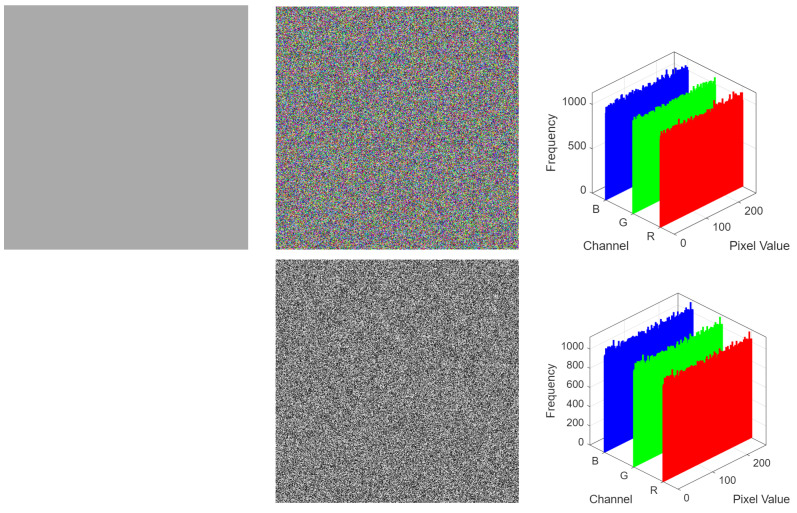
Cryptanalysis of the proposed encryption algorithm.

We encrypted the uniform-color test images using the proposed algorithm. As shown in Figure 12, the resulting ciphertext images exhibit snow-like randomness, with no visible patterns or structural biases, indicating strong confusion and diffusion effects. Experimental results affirm that the proposed scheme withstands chosen-plaintext attacks.

### 4.10. Run Time Analysis

To evaluate the computational efficiency of the proposed image encryption scheme, we conducted experiments on a PC equipped with an AMD 5950X processor, 128 GB RAM, and 8TB storage, using MATLAB R2025a. The experiments involved repeatedly encrypting standard RGB images of sizes 256 × 256 × 3 and 512 × 512 × 3, with the average execution time calculated over multiple iterations. The results are summarized in Table 6, which compares the proposed method against several recent schemes, including AES and state-of-the-art algorithms from 2024 and 2025. As demonstrated in Table 6, the proposed method significantly outperforms conventional encryption standards like AES in computational speed. These results clearly indicate that the proposed scheme is not only secure and effective but also highly efficient, making it suitable for real-time image encryption applications.

## 5. Conclusions

This paper proposes an image security communication enhancement scheme based on Fibonacci interleaved diffusion and non-degenerate discrete chaos. The scheme utilizes a hash function to extract the hash characteristic values of the plaintext image, generates an initial perturbation key-driven chaotic system to produce an initial pseudo-random sequence, and then applies a lightweight bit-level scrambling to the input image. The intermediate ciphertext image is then subjected to diffusion and random permutation using the Fibonacci Q matrix, resulting in the final ciphertext image. Throughout the encryption process, a plaintext correlation mechanism is employed. Consequently, due to the feedback loop of the plaintext, the algorithm is capable of resisting known-plaintext and chosen-plaintext attacks. Experimental results demonstrate that our proposed algorithm exhibits high security and robustness, with strong resistance to various cryptographic attacks. Consequently, the image encryption algorithm proposed in this paper represents a preferred security communication technology solution, with broad prospects for applications such as secure transmission of multimedia information in big data environments. 

## Figures and Tables

**Table 1:** Neighboring pixel correlation values for plaintext and ciphertext image in different directions.

Component	Direction	Original Image	Proposed
	Horizontal	0.9882	0.0010
R channel	Vertical	0.9821	0.0036
	Diagonal	0.9651	−0.0201
	Anti-diagonal	0.9741	0.0071
	Horizontal	0.9820	−0.0031
G channel	Vertical	0.9668	0.0059
	Diagonal	0.9556	−0.0471
	Anti-diagonal	0.9683	−0.0002
	Horizontal	0.9589	−0.0505
B channel	Vertical	0.9321	0.0175
	Diagonal	0.9158	−0.0121
	Anti-diagonal	0.9295	−0.0443

**Table 2:** Comparison of NPCR values between different algorithms.

Filename	Proposed	Ref. [56]	Ref. [57]	Ref. [58]	Ref. [59]
Airplane	99.6075	99.6283	99.6330	99.6092	/
Couple	99.6140	99.5845	/	/	99.6130
House	99.6017	99.6296	99.6399	99.6128	99.6110
Mandrill	99.6143	99.6296	/	99.6131	99.6110
Peppers	99.6178	99.6236	99.6174	99.6071	/
San Diego	99.6052	99.6291	99.6172	/	/
Tree	99.5926	99.6074	/	/	/
Female	99.6109	/	99.5880	/	/
Oakland	99.6088	/	99.6147	/	/
Stockton	99.6093	/	99.6066	/	/

**Table 3:** Comparison of information entropy between different algorithms.

Filename	Proposed	Ref. [56]	Ref. [57]	Ref. [58]	Ref. [59]
Airplane	7.9998	7.9983	7.9994	7.9992	/
Couple	7.9989	7.9987	/	/	7.9973
House	7.9989	7.9988	7.9978	7.9994	7.9968
Mandrill	7.9998	7.9986	/	7.9992	7.9992
Peppers	7.9998	7.9992	7.9994	7.9989	7.9971
San Diego	7.9998	7.9995	7.9998	/	/
Tree	7.9990	7.9994	/	/	/
Female	7.9990	/	7.9974	/	7.9971
Oakland	7.9999	/	7.9998	/	/
Stockton	7.9999	/	7.9998	/	/

**Table 4:** Results of the proposed encryption system: NPCR, UACI, BACI, MSE, PSNR, SSIM, information entropy.

Filename	Description	Size	Channel	NPCR	UACI	BACI	MSE	PSNR	SSIM	HI	HC
4.1.01	Female (NTSC test image)	256	Red	99.5789	32.0279	24.5693	36,350	2.5258	0.0094	6.8981	7.9992
4.1.01	Female (NTSC test image)	256	Green	99.6155	36.2976	27.4752					
4.1.01	Female (NTSC test image)	256	Blue	99.6384	37.4646	28.2366					
4.1.02	Couple (NTSC test image)	256	Red	99.6338	38.3150	28.5548	46,240	1.4806	0.0062	6.2945	7.9989
4.1.02	Couple (NTSC test image)	256	Green	99.6140	41.1581	30.5070					
4.1.02	Couple (NTSC test image)	256	Blue	99.6140	41.5659	30.7450					
4.1.03	Female (from Bell Labs?)	256	Red	99.6216	27.0281	19.0294	19,733	5.1789	0.0118	5.9709	7.9991
4.1.03	Female (from Bell Labs?)	256	Green	99.5850	26.6231	18.6229					
4.1.03	Female (from Bell Labs?)	256	Blue	99.5941	26.7814	18.8846					
4.1.04	Female	256	Red	99.6109	31.0961	23.4600	25,462	4.0718	0.0107	7.4270	7.9990
4.1.04	Female	256	Green	99.5911	30.6080	22.7991					
4.1.04	Female	256	Blue	99.6246	27.4894	19.8438					
4.1.05	House	256	Red	99.6323	27.3262	19.8229	25,063	4.1405	0.0098	7.0686	7.9989
4.1.05	House	256	Green	99.6338	29.9497	22.7503					
4.1.05	House	256	Blue	99.6017	31.3619	23.9620					
4.1.06	Tree	256	Red	99.6323	30.1875	22.9725	29,906	3.3732	0.0100	7.5371	7.9990
4.1.06	Tree	256	Green	99.6368	34.2251	26.7309					
4.1.06	Tree	256	Blue	99.5926	31.6424	24.5670					
4.1.07	Jelly beans	256	Red	99.6078	30.8726	24.4918	27,006	3.8162	0.0102	6.5835	7.9990
4.1.07	Jelly beans	256	Green	99.6246	32.5520	26.0160					
4.1.07	Jelly beans	256	Blue	99.5636	28.1269	21.1757					
4.1.08	Jelly beans	256	Red	99.6292	30.7943	24.3415	26,610	3.8803	0.0099	6.8527	7.9991
4.1.08	Jelly beans	256	Green	99.6490	31.8561	25.1864					
4.1.08	Jelly beans	256	Blue	99.6384	28.3174	21.0733					
4.2.01	Splash	512	Red	99.6136	34.2512	26.5957	33,716	2.8524	0.0100	7.2428	7.9998
4.2.01	Splash	512	Green	99.5983	35.6868	27.4607					
4.2.01	Splash	512	Blue	99.6109	31.9245	25.2354					
4.2.03	Mandrill (a.k.a. Baboon)	512	Red	99.6208	29.9609	22.3660	25,848	4.0065	0.0097	7.7624	7.9998
4.2.03	Mandrill (a.k.a. Baboon)	512	Green	99.6231	28.6218	21.6130					
4.2.03	Mandrill (a.k.a. Baboon)	512	Blue	99.6143	31.2127	23.9263					
4.2.05	Airplane (F-16)	512	Red	99.6143	31.9916	25.0842	31,045	3.2109	0.0099	6.6639	7.9998
4.2.05	Airplane (F-16)	512	Green	99.6075	33.0359	26.0583					
4.2.05	Airplane (F-16)	512	Blue	99.5869	32.7045	25.8280					
4.2.06	Sailboat on lake	512	Red	99.6140	27.9360	20.6481	30,347	3.3097	0.0105	7.7622	7.9998
4.2.06	Sailboat on lake	512	Green	99.5796	34.3896	26.7540					
4.2.06	Sailboat on lake	512	Blue	99.6254	34.3947	27.1026					
4.2.07	Peppers	512	Red	99.6223	28.9599	21.7661	30,327	3.3125	0.0104	7.6698	7.9998
4.2.07	Peppers	512	Green	99.6178	33.8983	25.9169					
4.2.07	Peppers	512	Blue	99.5857	33.7585	25.7967					
house	House	512	Red	99.6193	30.1928	23.1123	27,746	3.6989	0.0091	7.4858	7.9998
house	House	512	Green	99.6113	31.3346	24.0914					
house	House	512	Blue	99.6414	31.1798	23.9693					

**Table 5:** Plaintext sensitivity analysis of different images.

	Channel	(H/4, W/4)	(H × 3/4, W/4)	(H/4, W × 3/4)	(H×3/4, W × 3/4)
4.1.01	Red	99.5895	99.5941	99.6124	99.6338
4.1.01	Green	99.6353	99.6307	99.5956	99.5712
4.1.01	Blue	99.5972	99.6384	99.6124	99.5895
4.1.02	Red	99.5773	99.5926	99.6323	99.6246
4.1.02	Green	99.6307	99.6017	99.6185	99.6155
4.1.02	Blue	99.5804	99.5667	99.6414	99.6094
4.1.03	Red	99.6140	99.6414	99.6002	99.6246
4.1.03	Green	99.5667	99.5834	99.6094	99.6872
4.1.03	Blue	99.5682	99.5850	99.6155	99.5697
2.1.01	Red	99.6159	99.6067	99.6071	99.6017
2.1.01	Green	99.6307	99.6429	99.6101	99.5945
2.1.01	Blue	99.6216	99.6254	99.6082	99.6178
2.1.02	Red	99.6014	99.6193	99.6357	99.5781
2.1.02	Green	99.6166	99.6025	99.6170	99.6201
2.2.02	Red	99.6089	99.6164	99.6058	99.6119
2.2.02	Green	99.6180	99.6065	99.6064	99.6090
2.2.02	Blue	99.6103	99.6139	99.6077	99.6006
2.2.03	Red	99.6074	99.6130	99.6183	99.5976
2.2.03	Green	99.6119	99.6126	99.6103	99.6076
2.2.03	Blue	99.6099	99.6031	99.6061	99.6064

**Table 6:** Comparison of time with different algorithms.

Image Size	Time (s)				
	**Ref. [60]**	**Ref. [61]**	**Ref. [62]**	**AES [62]**	**Proposed**
512 × 512 × 3	-	0.9447	-	-	0.450793
256 × 256 × 3	0.858302	-	1.44177	75.0690	0.11241

## Data Availability

The datasets used and analyzed during the current study available from the corresponding author on reasonable request. All data generated or analyzed during this study are included in this article.
